# Cross-cultural adaptation of the "Australian National University Alzheimer's Disease Risk Index" for the Brazilian population

**DOI:** 10.1590/1980-57642016dn11-020009

**Published:** 2017

**Authors:** Marcus Kiiti Borges, Alessandro Ferrari Jacinto, Vanessa de Albuquerque Citero

**Affiliations:** 1 MSc Psychiatrist, Master’s student, Postgraduate Program in Psychiatry, Escola Paulista de Medicina, Universidade Federal de São Paulo (EPM/UNIFESP), São Paulo, SP, Brazil.; 2 MD Geriatrician, PhD Associate Professor, Department of Internal Medicine, Faculdade de Medicina de Botucatu, Universidade Estadual Paulista Júlio de Mesquita Filho (UNESP), Botucatu, SP, Brazil.; 3 MD Psychiatrist, PhD Associate Professor, Department of Psychiatry, Escola Paulista de Medicina, Universidade Federal de São Paulo (UNIFESP), São Paulo, SP, Brazil.

**Keywords:** Alzheimer's disease, dementia, risk assessment, primary prevention, cross-cultural comparison, doença de Alzheimer, demência, medição de risco, prevenção primária, comparação transcultural

## Abstract

Alzheimer's disease (AD) represents a major public health problem and it is therefore crucial that modifiable risk factors be known prior to onset of dementia in late-life. The "Australian National University - Alzheimer's Disease Risk Index" (ANU-ADRI) is one of the potential tools for primary prevention of the disease.

**Objective:**

The aim of this study was to devise an adapted version of the ANU-ADRI for use in Brazil.

**Methods:**

The instrument was translated from its original language of English into Portuguese and then back-translated into English by bilingual translators. It was subsequently reviewed and evaluated as to the degree of translation issues and equivalence. In this study, the ANU-ADRI was applied using individual (face-to-face) interviews in a public hospital, unlike the original version which is applied online by self-report. The final version (pretest) was evaluated in a sample of 10 participants with a mean age of 60 years (±11.46) and mean education of 11 years (±6.32).

**Results:**

The intraclass correlation coefficient (ICC) (inter-rater) was 0.954 (P<0.001 for a confidence interval (CI) of 95%=[0.932; 0.969]). Cultural equivalence was performed without the need for a second instrument application step.

**Conclusion:**

After cross-cultural adaptation, the language of the resultant questionnaire was deemed easily understandable by the Brazilian population.

## Abstract (pt)

A doença de Alzheimer (DA) tem sido um grande problema de saúde pública, portanto é crucial que fatores de risco modificáveis possam ser conhecidos antes da instalação da demência na fase final da vida. O "*Australian National University - Índice de Risco da Doença de Alzheimer*" (ANU-ADRI) é uma das ferramentas potenciais para a prevenção primária da doença.

**Objetivo:**

Este estudo teve como objetivo elaborar uma versão adaptada do ANU-ADRI para o uso no Brasil.

**Métodos:**

O instrumento foi traduzido do idioma original, inglês para a língua portuguesa e retrotraduzido para o inglês por tradutores bilíngues. Foi posteriormente revisado e avaliado quanto ao grau de dificuldade da tradução e equivalência. Neste estudo, o ANU-ADRI foi aplicado em entrevistas individuais (face a face) num hospital público em vez da versão original que é aplicada *on-line* por meio de autorrelato. A versão final (pré-teste) foi avaliada numa amostra de 10 participantes com média de idade 60 anos (±11,46), e média de escolaridade 11 anos (±6,32).

**Resultados:**

O coeficiente de correlação intraclasse entre os avaliadores foi 0,954 (P<0,001, intervalo de confiança de 95% IC=[0,932; 0,969]). A equivalência cultural foi realizada sem a necessidade de uma segunda etapa de aplicação do instrumento.

**Conclusão:**

Após a adaptação transcultural, a linguagem do questionário é descrita como de fácil compreensão pela população brasileira.

## INTRODUCTION

Alzheimer's disease (AD) is one of the most prominent public health issues. Around 8 million new cases of dementia are diagnosed each year, with AD being the most prevalent type.^1^ A recent meta-analysis has shown that the global prevalence of this form of dementia has doubled every 20 years in a number of countries with aging populations.^2^ The estimated global cost of the disease was US$ 604 billion in 2010, which is comparable to the economic burden of cancer and cardiovascular diseases.^3^

Multiple factors are associated with the risk of developing AD.^4^ The lifetime risk factors for AD may vary, with middle age representing a critical period for changes in some of these factors.^5^ While a number of clinical or lifestyle-related factors such as a low educational level (less than 12 years), diabetes, hypertension in middle age, obesity in middle age, depression, dyslipidemia, and smoking are modifiable, biological or genetic factors such as age, gender, and apolipoprotein (APOE) ε4 genotypes are not.^6^

The protective factors with stronger evidence are cognitive activities or reserve, physical activity, and engaging in other leisure activities that can stimulate social and cognitive aspects.^7^ Known protective factors are: Mediterranean diet (high in Omega-3 fatty acids) and moderate alcohol consumption, both lifestyle-related.^8^

Focusing on the risk of AD as opposed to diagnosis, the *Australian National University - Alzheimer's Disease Risk Index* (ANU-ADRI) could be used as one of several primary prevention strategies.^9^ The original items of the ANU-ADRI were developed in English, precluding their use in other countries such as Brazil. Thus, the literature recommends the process of translation and cross-cultural adaptation using rigorous and widely used methods.^12^

Finally, there are no papers published on cross-cultural adaptation of the ANU-ADRI for the Portuguese language. The instrument has not yet been used in specialized Neurology, Geriatrics or Psychiatry outpatient services or primary care (where patients are seen by general practitioners), and its applicability in Brazil has not yet been adequately assessed.Our objective in this study to devise an adapted version of the ANU-ADRI for use in Brazil.

## METHODS

**Ethical considerations.** Permission to translate and adapt the instrument was granted by the researchers at the Centre for Research on Ageing, Health and Wellbeing (CRAHW) of the Australian National University (ANU) and by Dr. Kaarin J. Anstey (Email: kaarin.anstey@anu.edu.au). This research project was reviewed and approved by the institutional review boards of the "*Universidade Federal de São Paulo*" - UNIFESP and "*Escola Paulista de Medicina*" - EPM (applying institution). Subsequently, the "*Centro de Educação em Saúde*"(Health Education Center) Research Ethics Committee of the Health Department of Curitiba approved the project regarding the feasibility of access to the research venue. The approved research project is available on the "*Plataforma Brasil*" database (CAAE registry No. 38185614.5.1001.5505).

Design: cross-cultural adaptation study

**Figure 1:**
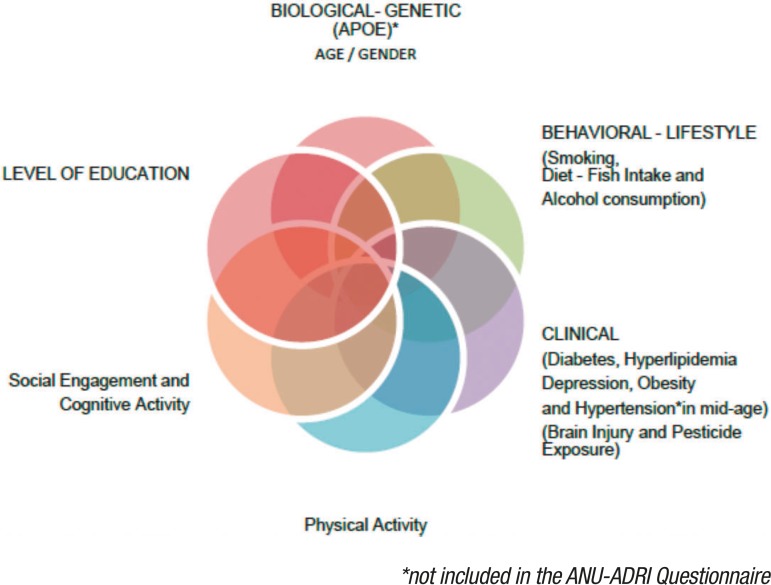
Risk and protective factors for AD (Source: adapted from Anstey et al.^9^).

**Instruments.** A questionnaire was administered for sociodemographic and clinical data collection, after which the MMSE was applied for patient screening. The 84-item ANU-ADRI was the principal questionnaire. The Australian National University Alzheimer's Disease Risk Index (ANU-ADRI) is a self-report instrument to assess: 11 risk factors and 4 protective factors for Alzheimer's disease. These factors are shown in Figure 1.

The total score was obtained by summing the scores assigned by category to the risk or protective factors in Table 1. In this study, the ANU-ADRI was administered via individual face-to-face interviews as opposed to applying the original online self-report version.

**Table 1:** Variables assessed and scores allocated to each factor on the ANU-ADRI (Anstey et al.^10^).

• Age and gender (Male<65=0; Female<65=0) : self-report (sociodemographic characteristics).
• Education (>11=0; 8 to 11=3; <8=6) : scale was created using number of years.
• BMI (Normal=0; Overweight=2; Obese=5): BMI >25 or >30 were used as cut-offs.
• High cholesterol (No=0; Yes=3): self-report (history of hyperlipidemia).
• Diabetes (No=0; Yes=3): self-report (history of diabetes and medication).
• TBI (No=0; Yes=4): self-report (history for TBI with loss of consciousness).
• Depression (CES-D<16=0; CES-D>16=2) : CES-D> 16 was used as cut-off.
• Physical activity (Mild=0; Moderate=-2; Vigorous=-3): IPAQ categories were used with MET.
• Cognitive activities (Low=0; Moderate=-7; High=-6): focused on cognitive activities in late life.
• Social engagement (Low=6; Low to Moderate=4; Moderate to High=1; High=0): self-report.
• Fish intake (0 to 0.25=0; 0.26 to 2=-3; 2.1 to 4=-4; ≥ 4.1=-5): National Cancer Institute FFQ.
• Alcohol consumption (None=0; Light to Moderate=-3): NHMRC guidelines used.
• Smoking (Current=4; Past=1; Never=0): self-report (questions for smoking status).
• Pesticide Exposure (No=0; Yes=2): self-report (history of pesticide exposure).

BMI: Body Mass Index; TBI: Traumatic Brain Injury; CES-D: Center for Epidemiology Scale for Depression; IPAQ: International Physical Activity Questionnaire; MET: Metabolic Equivalent; FFQ: Food Frequency Questionnaire; MHMRC: Australian National and Medical Research Council.

**Participants.** A convenience sample comprised 10 patients selected from a public hospital in Curitiba, Brazil, between July and August 2015. All participants were aged >40 years with no evidence of severe or disabling disease at the outpatient service of "Hospital do Idoso Zilda Arns" (HIZA).

The exclusion criteria were a history of any severe visual or hearing impairment or marked psychomotor disability (e.g. parkinsonism); a history of severe psychiatric or neurological disorders; severe clinical or psychiatric disorders; dementia and/or below expected score on the Mini-Mental State Examination (MMSE) adjusted for level of education (cut-off scores: 20 for illiterate persons; 25 for those with at least one year of schooling).^11^ No patients were excluded from the study. All patients were instructed as to the study goals and provided written informed consent prior to instrument evaluation.

**Procedures.** The translation and cross-cultural adaptation were standardized.^12^ The first step was a forward translation of the English version of the ANU-ADRI instrument into Brazilian Portuguese by two bilingual health professionals (DI and RNS), both of whom were Brazilian, independent, and aware of the main goal of the study. Translation focused on conceptual rather than linguistic equivalence of the items. The translations were compared by an expert review committee (two psychiatrists, MKB and VAC, and one geriatrician, AFJ) and a consensus version was formulated from these two translations.

In the next step, the consensus translation was back-translated into English by a third bilingual translator - a native English speaker with proficiency in the Portuguese language (ACD). Subsequently, this version was compared with the original English language instrument. The back-translation showed semantic equivalence and consistency in the translated items.

The final step consisted of ensuring cultural equivalence and involved the same expert panel who participated in the semantic equivalence process. This step is justified by the fact that a word or statement used with a given intent in the original context may not convey the same meaning to the target population of the new version. The expert committee reviewed the questions with regard to content validity and prepared a summary version for pretesting in order to assess the acceptability and understanding of the instrument by the target population. The aim of this step was to identify the questions that were not understood or not answered by the target population. To that end, a "not applicable" answer choice was included alongside each question. The ANU-ADRI instrument was administered by five trained interviewers yielding data in the pretest phase.

**Statistical analysis.** When evaluating screening instruments, test indices may be inaccurate if samples are too small. The aim of testing reproducibility is to assess the random fluctuations noted for the same individual interviewed on multiple occasions. Each patient was assessed by a different interviewer in a random manner (inter-rater).

Intraclass correlation coefficient (ICC) was the specific method used to estimate inter-rater reliability, with values around 1 indicating good agreement between answers.^13^ ICC is defined as "the proportion of variance of an observation due to between-subject variability in true scores" and in order to offset potential errors, a 95% confidence interval or standard error should be adopted.^14^ The data were analyzed using the SPSS version 20.0 statistical package.

## RESULTS

Table 2 shows the sociodemographic characteristics. Mean pretest MMSE and ANU-ADRI scores of the sample are given in Table 3.

**Table 2:** Sociodemographic characteristics of the sample.

Sociodemographic characteristics	Categories (%)	Participants (n=10)	Mean (SD)	Range (years)
**Age**			60 (11.46)	(41-77)
**Gender**	Male (50%)	(n=5)		
	Female (50%)	(n=5)		
**Education**			11 (6.32)	(0-18)
**Marital status**	Married (40%)	(n=4)		
	Never Married (30%)	(n=3)		
	Widowed (20%)	(n=2)		
	Separated (10%)	(n=1)		
**Occupation**	Retired (60%)	(n=6)		
	Qualified worker (20%)	(n=2)		
	Unemployed (20%)	(n=2)		

SD: standard deviation.

**Table 3:** Mean pretest MMSE and ANU-ADRI scores.

	Mean	SD	Minimum	Maximum
MMSE	25.6	2.87	20	30
ANU-ADRI	6.3	13.8	-12	37

MMSE: Mini-Mental State Examination; ANU-ADRI: Australian National University Alzheimer's Disease Risk Index; SD: standard deviation.

Cross-cultural equivalence was achieved with no need for a second step in the instrument evaluation process. The intra-class correlation coefficient (inter-rater) of the ANU-ADRI was 0.954 (*p*<0.001, 95% CI=[0.932; 0.969]).

Most of the individuals interviewed (90%) needed the interviewer's assistance to read the items concerning "Physical Activity" and "Depression" domains, yet had no difficulty choosing the answers. None of the 84 questions were deemed "not applicable" by the respondents.

The mean time for administering the ANU-ADRI was 25 (±5) minutes. The final adapted Portuguese version can be found as (*Supplementary material*).

## DISCUSSION

The cross-cultural adaptation process is a complex task, as it entails following a series of steps until functional equivalence is achieved. The most adequate adapted version for the older Brazilian population underwent minor changes in the items concerning the Physical Activity domain based on the International Physical Activity Questionnaire (IPAQ) scale adapted for older Brazilian individuals^15^ and validated for older women^16^ and men.^17^

Cultural equivalence presupposes a literal match between the original and adapted versions, and should address the impact a term would have in the cultural setting of the target population sample. The expressions used in item 50 ("shoveling snow") and item 60 ("doubles tennis") of the source questionnaire were excluded following cultural adaptation as they would not be as meaningful in the adapted version for the Brazilian population. To allow the use of IPAQ-based questions for older individuals, examples were included of activities that are common for persons in this age group.

Other expressions from the Brazilian version of the IPAQ validated for older men^16^ were included without changing the original structure of the questionnaire - for example, in items 52 ("limpar a garagem"), 54 ("lavar roupas à mão, limpar o banheiro"), 58 ("remo, canoagem, musculação ou esportes em geral"), and 60 ("jogar bola, praticar hidroginástica, ginástica ou dança").

It is important to know the particularities of the sample considering the fact that respondents' gender, age group, and level of education could have influenced the performance of the instrument. Older adults with a low educational level had more difficulty understanding the items related to the "Depression" domain based on the CES-D scale validated in Brazil^18^ compared to adults with a higher level of education.

A randomized, controlled study including a sample of middle-aged adults (mean age=55 years) with a high educational level (mean=18 years of education) revealed a lower mean ANU-ADRI score (-1.38) than that found in our sample (6.3).^19^ The author of the cited study noted that the ANU-ADRI should be tested in "target samples with lower levels of education and higher ANU-ADRI risk scores".

In the present study, the mean administration time was longer (25±5 minutes) than that of the original ANU-ADRI, reported to be around 15±5 minutes. The short version of the ANU-ADRI can be considered an alternative whenever the original version is deemed too lengthy or "not applicable" due to limitations in administration time.^20^ The assessment of older people via questionnaires (either self-administered or in the form of interviews) is a particularly difficult task due to the inaccuracy of the information given and susceptibility to recording or recall bias. In the present study, the ANU-ADRI was administered using individual interviews as opposed to the self-report approach of the original online version. The original version, the short form and the Portuguese version are available for printout from the internet.^21^

In conclusion, after the cross-cultural adaptation of the ANU-ADRI, the wording of the instrument was found to be easily understandable by the Brazilian population.

Furthermore, the final adapted version can facilitate international cooperation projects that employ this instrument.

In the near future, the authors of the present study intend to publish the validity and test-retest reliability of the ANU-ADRI with a larger sample (n=100) of participants from the same institution in Brazil.

## SUPPLEMENTARY MATERIAL. BRAZILIAN PORTUGUESE VERSION OF THE ANU-ADRI QUESTIONNAIRE

**Table:**

**Parte 1 - SOBRE VOCÊ**					
**Primeiro, gostaríamos de solicitar algumas informações básicas e história pessoal. Por gentileza, faça um círculo em volta das respostas apropriadas.**					
1.	Quantos anos você completou no último aniversário?		___	Anos	
2.	Qual é a sua data de nascimento?		___	Ano	
			___	Mês	
3.	Qual é o seu sexo?		1	Masculino	
			2	Feminino	
4.	Qual a sua escolaridade? (escreva o número de anos em cada nível)		___	Ensino Fundamental	
			___	Ensino Médio	
			___	Escola Técnica	
			___	Universidade	
			___	Outros	
5.	Qual é o seu estado civil atual?		1	Casado(a)	
			2	União Estável	
			3	Separado(a)	
			4	Divorciado(a)	
			5	Viúvo(a)	
			6	Solteiro(a)	
6.	Pode nos informar sua altura?		___	Centímetros	
			9	Não sei	
7.	Qual o seu peso sem as roupas e sapatos?		___	Quilogramas	
			9	Não sei	
**Parte 2 - SOBRE A SUA SAÚDE**					
**Esta seção se refere aos seus problemas de saúde.**					
8.	Você está ciente dos seus níveis de colesterol totais? (nos últimos 2 anos)		___	Colesterol Total	
9.	Nos últimos dois anos, algum médico ou outro profissional de saúde lhe disse que você tem colesterol alto?		1	Sim	
			2	Não	
			3	Não sei	
10.	Alguma vez um médico ou outro profissional de saúde lhe disse que você tem diabetes?		1	Sim	
			2	Não	
			3	Não sei	
11.	Alguma vez um médico ou outro profissional de saúde lhe disse que você tem níveis altos de açúcar no sangue ou na urina?		1	Sim	
			2	Não	
			3	Não sei	
**!**	**SE A RESPOSTA FOR NÃO, PASSE PARA A QUESTÃO 13**				
12.	Que tratamento para diabetes você está fazendo atualmente?		1	Insulina	
			2	Insulina e comprimidos	
			3	Comprimidos	
			4	Apenas dieta	
			5	Nenhum	
			6	Outro	
13.	Você já teve ferimentos na cabeça?		1	Sim	
			2	Não	
			3	Não sei	
**!**	**SE A RESPOSTA FOR NÃO, PASSE PARA A QUESTÃO 16**				
14.	Considerando o ferimento mais grave que você teve na cabeça, você chegou a perder a consciência?		1	Sim	
			2	Não	
			3	Não sei	
15.	Em caso afirmativo, por quanto tempo você ficou inconsciente?		1	0-15 minutos	
			2	15-30 minutos	
			3	30 minutos a uma hora	
			4	Horas	
			5	Dias	
			6	Não sei	
16.	Alguma vez um médico ou outro profissional de saúde lhe disse que você tem depressão?		1	Sim	
			2	Não	
			3	Não sei	
As próximas perguntas são sobre seus sentimentos. Para cada uma das afirmativas abaixo, pedimos que informe se você se sentiu assim **durante a semana que passou. ** **As alternativas são:** 0 Raramente ou em nenhum momento (menos de um dia) 1 Por algum tempo ou um curto período (1-2 dias) 2 Ocasionalmente ou por um período razoável de tempo (3-4 dias) 3 A maior parte ou todo o tempo (5-7 dias) **Faça um círculo em volta da melhor resposta para cada pergunta.**					
		**Menos de** **1 dia**	**1-2** **dias**	**3-4** **dias**	**5-7** **dias**
17.	Senti-me incomodado com coisas que habitualmente não me incomodam.	**0**	**1**	**2**	**3**
18.	Não tive vontade de comer, tive pouco apetite.	**0**	**1**	**2**	**3**
19.	Senti não conseguir melhorar meu estado de ânimo mesmo com a ajuda de familiares e amigos.	**0**	**1**	**2**	**3**
20.	Senti-me tendo tanto valor quanto outras pessoas.	**0**	**1**	**2**	**3**
21.	Senti dificuldade em me concentrar no que estava fazendo.	**0**	**1**	**2**	**3**
22.	Senti-me deprimido.	**0**	**1**	**2**	**3**
23.	Senti que tive de fazer esforço para dar conta das minhas tarefas habituais.	**0**	**1**	**2**	**3**
24.	Senti-me otimista com relação ao futuro.	**0**	**1**	**2**	**3**
25.	Considerei que minha vida tinha sido um fracasso.	**0**	**1**	**2**	**3**
26.	Senti-me amedrontado.	**0**	**1**	**2**	**3**
27.	Meu sono não foi repousante.	**0**	**1**	**2**	**3**
28.	Estive feliz.	**0**	**1**	**2**	**3**
29.	Falei menos do que o habitual.	**0**	**1**	**2**	**3**
30.	Senti-me sozinho.	**0**	**1**	**2**	**3**
31.	As pessoas não foram amistosas comigo.	**0**	**1**	**2**	**3**
32.	Aproveitei a minha vida.	**0**	**1**	**2**	**3**
33.	Tive crises de choro.	**0**	**1**	**2**	**3**
34.	Senti-me triste.	**0**	**1**	**2**	**3**
35.	Eu senti que as pessoas não gostavam de mim.	**0**	**1**	**2**	**3**
36.	Não consegui levar adiante minhas coisas.	**0**	**1**	**2**	**3**
**Parte 3 - SOBRE A SUA ATIVIDADE**					
As próximas perguntas referem-se ao tempo em que você esteve fisicamente ativo(a) nos **últimos 7 dias.** Pense em todas as atividades **vigorosas ** e **moderadas ** que você fez **na semana que passou. ** Atividades físicas **vigorosas ** são aquelas que exigem bastante esforço físico e fazem você respirar muito mais rápido que o normal. Atividades **moderadas ** são aquelas que exigem esforço físico moderado e fazem você respirar um pouco mais rápido que o normal.					
**PARTE 3a: ATIVIDADE FÍSICA NO TRABALHO**					
A primeira seção é sobre o seu trabalho. Ela inclui serviços remunerados, atividades agrícolas, trabalho voluntário, atividades nos estudos e qualquer outro trabalho não remunerado que você fez fora de casa. Não inclua trabalho não remunerado que você faz em casa, como tarefas domésticas, cuidar do quintal, manutenção geral da casa e cuidar da família.					
37.	Atualmente, você tem um trabalho remunerado ou faz algum trabalho voluntário fora de casa?		1	Sim	
			2	Não	
!	**SE A RESPOSTA FOR NÃO, PASSE PARA A PARTE 3b - TRANSPORTE**				
As próximas perguntas são sobre toda a atividade física que você fez na **última semana ** como parte do seu trabalho, remunerado ou não. Isso não inclui as idas e vindas do trabalho.					
38.	Em quantos dias da **última semana ** você praticou atividades físicas **vigorosas ** como erguer grandes pesos, cavar, trabalhar em obras ou subir escadas como **parte do seu trabalho** ? Considere apenas as atividades físicas que você fez por pelo menos 10 minutos seguidos.		___	Dias por semana	
			0	Nenhuma atividade vigorosa no trabalho	
!	**SE NÃO REALIZOU ATIVIDADE VIGOROSA, PASSE PARA A QUESTÃO 40**				
39.	Nos dias em que você fez atividades físicas **vigorosas** como parte do seu trabalho, quanto tempo você gastou nessas atividades, em geral?		___	Horas por dia	
			___	Minutos por dia	
40.	Considere mais uma vez apenas as atividades físicas que você praticou por pelo menos 10 minutos seguidos. Em quantos dias da **última semana ** você praticou atividades físicas **moderadas** , tais como carregar pesos leves, **como parte do seu trabalho** ? Não inclua caminhadas.		___	Dias por semana	
			0	Nenhuma atividade física moderada no trabalho	
!	**SE NÃO REALIZOU ATIVIDADE FÍSICA MODERADA, PASSE PARA A QUESTÃO 42**				
41.	Nos dias em que você fez atividades físicas **moderadas ** como parte do seu trabalho, quanto tempo você gastou nessas atividades, em geral?		___	Horas por dia	
			___	Minutos por dia	
42.	Em quantos dias da **última semana ** você **caminhou ** por pelo menos 10 minutos seguidos **como parte do seu trabalho** ? Não inclua caminhadas para ir ou voltar do trabalho.		___	Dias por semana	
			0	Nenhuma caminhada	
!	**SE VOCÊ NÃO CAMINHOU, PASSE PARA A QUESTÃO 44**				
43.	Nos dias em que você **caminhou ** como parte do seu trabalho, quanto tempo você gastou caminhando, em geral?		___	Horas por dia	
			___	Minutos por dia	
**PARTE 3b: ATIVIDADE FÍSICA NO TRANSPORTE**					
Estas perguntas se referem a como você se deslocou de um lugar para outro, incluindo locais como: trabalho, lojas, cinema, supermercado, etc.					
44.	Em quantos dias da **última semana ** você usou um veículo motorizado, como carro, ônibus, metrô, trem ou outro, como meio de transporte?		___	Dias por semana	
			0	Nenhum deslocamento em veículo motorizado	
!	**SE NÃO USOU TRANSPORTE MOTORIZADO, PASSE PARA A QUESTÃO 46**				
45.	Nos dias em que você se deslocou usando carro, ônibus, metrô, trem ou outro tipo de veículo motorizado, quanto tempo você gastou nesse deslocamento?		___	Horas por dia	
			___	Minutos por dia	
46.	Em quantos dias da **última semana ** você pedalou por pelo menos 10 minutos seguidos para ir de um lugar para outro?		___	Dias por semana	
			0	Nenhum uso de bicicleta	
!	**SE NÃO USOU BICICLETA, PASSE PARA A QUESTÃO 48**				
47.	Em geral, quanto tempo você gastou em um desses dias para ir de bicicleta de um lugar para outro?		___	Horas por dia	
			___	Minutos por dia	
48.	Em quantos dias da **última semana ** você **caminhou ** por pelo menos 10 minutos seguidos para se deslocar de um lugar para outro?		___	Dias por semana	
			0	Nenhuma caminhada	
!	**SE NÃO CAMINHOU, PASSE PARA A QUESTÃO 50**				
49.	Em geral, quanto tempo você gastou em um desses dias em que caminhou de um lugar para outro?		___	Horas por dia	
			___	Minutos por dia	
**PARTE 3c: TAREFAS DOMÉSTICAS, MANUTENÇÃO DA CASA E CUIDADOS COM A FAMÍLIA**					
Esta seção se refere a algumas das atividades físicas que você pode ter realizado em sua casa nos últimos 7 dias, tais como tarefas domésticas, jardinagem, trabalhar no quintal, fazer trabalhos gerais de manutenção da casa e cuidar da família.					
50.	Considere mais uma vez apenas as atividades físicas que você fez por pelo menos 10 minutos seguidos. Em quantos dias da **última semana ** você realizou atividades físicas **vigorosas ** como erguer grandes pesos, cortar lenha ou cavar **no jardim ou quintal** ?		___	Dias por semana	
			0	Nenhuma atividade vigorosa	
!	**SE NÃO FEZ ATIVIDADE VIGOROSA, PASSE PARA A QUESTÃO 52**				
51.	Nos dias em que você fez atividades físicas **vigorosas ** no **jardim ou quintal, ** quanto tempo você gastou, em geral, nessas atividades?		___	Horas por dia	
			___	Minutos por dia	
52.	Considere mais uma vez apenas as atividades físicas que você realizou por pelo menos 10 minutos seguidos. Em quantos dias da **última semana ** você fez atividades **moderadas** , tais como carregar pesos leves, limpar a garagem e limpar o **jardim ou quintal** ?		___	Dias por semana	
			0	Nenhuma atividade moderada	
53.	Nos dias em que você realizou atividades físicas **moderadas ** no **jardim ou quintal** , quanto tempo você gastou, em geral, nessas atividades?		___	Horas por dia	
			___	Minutos por dia	
54.	Considere mais uma vez apenas as atividades físicas que você realizou por pelo menos 10 minutos seguidos. Em quantos dias da **última semana ** você realizou atividades **moderadas** , tais como limpar vidros ou janelas, lavar roupas à mão, limpar banheiro, esfregar ou varrer o chão **dentro de sua casa** ?		___	Dias por semana	
			0	Nenhuma atividade moderada	
!	**SE NÃO REALIZOU ATIVIDADE MODERADA, PASSE PARA A QUESTÃO 56**				
55.	Nos dias em que você realizou atividades físicas moderadas dentro de casa, quanto tempo você gastou, em geral, nessas atividades?		___	Horas por dia	
			___	Minutos por dia	
**PARTE 3d: RECREAÇÃO, ESPORTE e ATIVIDADE FÍSICA COMO LAZER**					
Esta seção é sobre todas as atividades físicas que você fez nos últimos 7 dias apenas para recreação, esporte, exercício ou lazer. Não inclua atividades que você já mencionou.					
56.	Sem contar as caminhadas que você já mencionou, em quantos dias da última semana você **caminhou ** por pelo menos 10 minutos seguidos nas suas horas de lazer?		___	Dias por semana	
			0	Nenhuma caminhada	
!	**SE NÃO CAMINHOU, PASSE PARA A QUESTÃO 58**				
57.	Nos dias em que você **caminhou ** nas suas horas de lazer, quanto tempo você gastou, em geral, nessa atividade?		___	Horas por dia	
			___	Minutos por dia	
58.	Considere mais uma vez apenas as atividades físicas que você realizou por pelo menos 10 minutos seguidos. Em quantos dias da **última semana ** você realizou atividades físicas **vigorosas ** como exercícios aeróbicos (corrida, pedalar ou nadar em ritmo acelerado, canoagem, remo, musculação ou esportes em geral) **nas suas horas de lazer** ?		___	Dias por semana	
			0	Nenhuma atividade vigorosa	
!	**SE NÃO REALIZOU ATIVIDADE VIGOROSA, PASSE PARA A QUESTÃO 60**				
59.	Nos dias em que você realizou atividades físicas **vigorosas ** nas suas horas de lazer, quanto tempo você gastou, em geral, nessas atividades?		___	Horas por dia	
			___	Minutos por dia	
60.	Considere mais uma vez apenas as atividades físicas que você realizou por pelo menos 10 minutos seguidos. Em quantos dias da **última semana ** você realizou atividades físicas **moderadas ** como pedalar ou nadar num ritmo regular ou jogar bola, praticar hidroginástica, ginástica ou dança **nas suas horas de lazer** ?		___	Dias por semana	
			0	Nenhuma atividade moderada	
!	**SE NÃO REALIZOU ATIVIDADE MODERADA, PASSE PARA A QUESTÃO 62**				
61.	Nos dias em que você realizou atividades físicas moderadas nas suas horas de lazer, quanto tempo você gastou, em geral, nessas atividades?		___	Horas por dia	
			___	Minutos por dia	
**Parte 4 - SOBRE SUAS HORAS DE LAZER**					
As perguntas seguintes serão sobre suas atividades de lazer.					
62.	Aproximadamente quanto tempo você passa **lendo ** diariamente, incluindo leituras *online* ?		1	Nenhum	
			2	Menos de uma hora	
			3	De uma até menos de duas horas	
			4	De duas a menos de três horas	
			5	Três ou mais horas	
			9	Não sei	
63.	Considerando o **último ano** , com que frequência você leu jornais, inclusive *online* ?		5	Todos os dias, ou quase todos os dias	
			4	Várias vezes por semana	
			3	Várias vezes por mês	
			2	Várias vezes por ano	
			1	Uma vez por ano ou menos	
			9	Não sei	
64.	Durante o **último ano** , com que frequência você leu revistas, inclusive *online* ?		5	Todos os dias, ou quase todos os dias	
			4	Várias vezes por semana	
			3	Várias vezes por mês	
			2	Várias vezes por ano	
			1	Uma vez por ano ou menos	
			9	Não sei	
65.	Durante o **último ano** , com que frequência você leu livros?		5	Todos os dias, ou quase todos os dias	
			4	Várias vezes por semana	
			3	Várias vezes por mês	
			2	Várias vezes por ano	
			1	Uma vez por ano ou menos	
			9	Não sei	
66.	Durante o **último ano** , com que frequência você jogou damas ou outros jogos de tabuleiro, cartas, fez quebra-cabeças, jogos de palavras, desafios, ou qualquer outro jogo semelhante? (incluindo jogos *online* )		5	Todos os dias, ou quase todos os dias	
			4	Várias vezes por semana	
			3	Várias vezes por mês	
			2	Várias vezes por ano	
			1	Uma vez por ano ou menos	
			9	Não sei	
67.	Durante o **último ano** , com que frequência você fez atividades para exercitar o cérebro?		5	Todos os dias, ou quase todos os dias	
			4	Várias vezes por semana	
			3	Várias vezes por mês	
			2	Várias vezes por ano	
			1	Uma vez por ano ou menos	
			9	Não sei	
68.	Durante o **último ano** , com que frequência você escreveu cartas ou *e-mails* ?		5	Todos os dias, ou quase todos os dias	
			4	Várias vezes por semana	
			3	Várias vezes por mês	
			2	Várias vezes por ano	
			1	Uma vez por ano ou menos	
			9	Não sei	
69.	Durante o **último ano** , com que frequência você usou redes sociais como o *Facebook * ou *Twitter* ?		5	Todos os dias, ou quase todos os dias	
			4	Várias vezes por semana	
			3	Várias vezes por mês	
			2	Várias vezes por ano	
			1	Uma vez por ano ou menos	
			9	Não sei	
70.	No **último ano** , quantas vezes você visitou um museu?		5	Todos os dias, ou quase todos os dias	
			4	Várias vezes por semana	
			3	Várias vezes por mês	
			2	Várias vezes por ano	
			1	Uma vez por ano ou menos	
			9	Não sei	
71.	No **último ano** , quantas vezes você assistiu a um concerto, peça teatral ou musical?		5	Todos os dias, ou quase todos os dias	
			4	Várias vezes por semana	
			3	Várias vezes por mês	
			2	Várias vezes por ano	
			1	Uma vez por ano ou menos	
			9	Não sei	
72.	No **último ano** , com que frequência você foi à biblioteca?		5	Todos os dias, ou quase todos os dias	
			4	Várias vezes por semana	
			3	Várias vezes por mês	
			2	Várias vezes por ano	
			1	Uma vez por ano ou menos	
			9	Não sei	
**Parte 5 - SOBRE OS SEUS AMIGOS E FAMÍLIA**					
Gostaríamos de saber sobre seus amigos e parentes. Considerando todos os seus amigos, incluindo os que moram na vizinhança:					
73.	Quantos de seus amigos e parentes você vê ou tem notícia no mínimo uma vez por mês?		1	Nenhum	
			2	Um	
			3	Dois	
			4	Três ou quatro	
			5	Cinco a oito	
			6	Nove ou mais	
74.	Você está satisfeito(a) com suas relações com amigos e parentes?		0	Sim	
			1	Não	
75.	Com que frequência você participa de cerimônias religiosas ou grupos sociais, políticos ou comunitários?		0	Menos do que uma vez na semana	
			1	Uma vez na semana ou mais vezes	
76.	Você mora sozinho(a) ou com outras pessoas?		0	Moro sozinho(a) ou com esposo(a)	
			1	Moro com família estendida (irmãos ou filhos e netos)	
**Parte 6 - SOBRE SUA COMIDA, BEBIDA e HÁBITOS**					
Nos últimos 12 meses:					
77.	Com que frequência você comeu peixe ou frutos do mar defumados (tais como salmão, ostras, truta ou outros)?		1	Nunca	
			2	1-6 vezes por ano	
			3	7-11 vezes por ano	
			4	1 vez por mês	
			5	2-3 vezes por mês	
			6	Uma vez por semana	
			7	Duas vezes por semana	
			8	3-4 vezes por semana	
			9	5-6 vezes por semana	
			10	Uma vez por dia	
			11	Duas ou mais vezes por dia	
78.	Com que frequência você comeu sushi ou sashimi (contendo peixe cru ou frutos do mar, inclusive mariscos)?		1	Nunca	
			2	1-6 vezes por ano	
			3	7-11 vezes por ano	
			4	1 vez por mês	
			5	2-3 vezes por mês	
			6	Uma vez por semana	
			7	Duas vezes por semana	
			8	3-4 vezes por semana	
			9	5-6 vezes por semana	
			10	Uma vez por dia	
			11	Duas ou mais vezes por dia	
79.	Com que frequência você comeu ostras cruas, mariscos crus ou outro tipo de peixe cru (sem contar o peixe cru no sushi)?		1	Nunca	
			2	1-6 vezes por ano	
			3	7-11 vezes por ano	
			4	1 vez por mês	
			5	2-3 vezes por mês	
			6	Uma vez por semana	
			7	Duas vezes por semana	
			8	3-4 vezes por semana	
			9	5-6 vezes por semana	
			10	Uma vez por dia	
			11	Duas ou mais vezes por dia	
80.	Com que frequência você comeu todos os outros tipos de peixe ou frutos do mar (incluindo mariscos) de outra forma que não fritos, defumados ou crus?		1	Nunca	
			2	1-6 vezes por ano	
			3	7-11 vezes por ano	
			4	1 vez por mês	
			5	2-3 vezes por mês	
			6	Uma vez por semana	
			7	Duas vezes por semana	
			8	3-4 vezes por semana	
			9	5-6 vezes por semana	
			10	Uma vez por dia	
			11	Duas ou mais vezes por dia	
As próximas perguntas tratam de seu consumo de álcool. Exemplos de dose-padrão:					

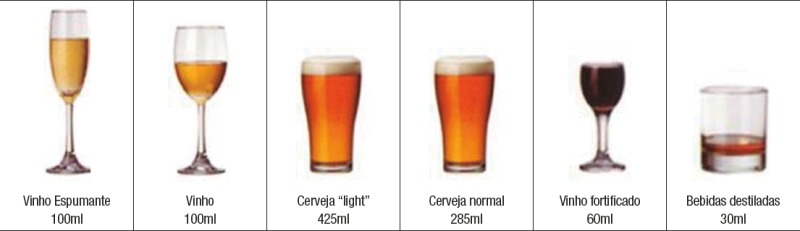

81.	Com que frequência você ingere bebida alcoólica?		0	Nunca	
			1	Uma vez ao mês ou menos	
			2	2-4 vezes ao mês	
			3	2-3 vezes na semana	
			4	4 ou mais vezes na semana	
!	**SE NÃO INGERE ÁLCOOL, PASSE PARA A QUESTÃO 83**				
82.	Quantas doses de bebida você consome num dia típico quando está bebendo?		0	1-2	
			1	3-4	
			2	5-6	
			3	7-9	
			4	10 ou mais	
A pergunta seguinte é sobre o uso de tabaco ou produtos com nicotina.					
83.	Você fuma ou fumou cigarros, charutos, cachimbo ou outros produtos do tabaco?		1	Sim, atualmente	
			2	Sim, não atualmente	
			3	Nunca	
A próxima pergunta se refere à sua exposição a toxinas.					
84.	Você já participou da mistura, aplicação ou carregamento de pesticidas, herbicidas, fumegantes ou fungicidas?		1	Sim	
			2	Não	
			8	Recuso-me a responder	
			9	Não sei	
